# Endoscopic Ultrasound in the Diagnosis of Extrahepatic Cholangiocarcinoma: What Do We Know in 2023?

**DOI:** 10.3390/diagnostics13061023

**Published:** 2023-03-08

**Authors:** Rares Ilie Orzan, Cristina Pojoga, Renata Agoston, Radu Seicean, Andrada Seicean

**Affiliations:** 1 Department of Internal Medicine, Iuliu Hațieganu University of Medicine and Pharmacy, 400347 Cluj-Napoca, Romania; 2 Regional Institute of Gastroenterology and Hepatology “Prof. Dr. Octavian Fodor”, 400162 Cluj-Napoca, Romania; 3 UBB Med, Babes-Bolyai University, 400347 Cluj-Napoca, Romania; 4 Faculty of Medicine, Iuliu Hatieganu University of Medicine and Pharmacy, 400347 Cluj-Napoca, Romania; 5 First Department of Surgery, Iuliu Hațieganu University of Medicine and Pharmacy, 400000 Cluj-Napoca, Romania

**Keywords:** extrahepatic cholangiocarcinoma, endoscopic ultrasound, endoscopic retrograde cholangiopancreatography

## Abstract

Extrahepatic cholangiocarcinoma (CCA) is a rare and aggressive type of cancer, presenting as a mass or as a biliary stricture. This review summarizes the utility of endoscopic ultrasound (EUS) in the detection, staging, and determination of the differential diagnosis, especially when no cause of bile duct dilatation is revealed by cross-sectional imaging. The EUS detection rate for distal CCAs is higher than that for the proximal CCAs. The accuracy of T staging varies between 60 and 80%, and vascular involvement is correctly assessed by conventional EUS. EUS-tissue acquisition from the primary tumors is reserved for unresectable or metastatic CCA, especially in distal strictures or mass CCAs. For proximal lesions, EUS could be performed as an adjunctive to ERCP sampling when the latter is inconclusive. EUS is not appropriate for assessing the malignant features of lymph nodes in CCAs. Lymph node EUS-tissue acquisition should be performed only if it changes the surgical decision. Perhaps the development of EUS-fine needle biopsy and the detection of molecular genetic alteration will increase the diagnostic yield in CCAs.

## 1. Introduction

Biliary tract cancer comprises a variety of invasive adenocarcinomas, including extrahepatic and intrahepatic cholangiocarcinoma (CCA), gallbladder carcinoma, and ampullary carcinoma [1]. Although CCA is a rare type of cancer, it is the second-most frequent primary tumor of the liver and the most frequent biliary malignancy, accounting for about 3% of all gastro-intestinal neoplasia [2]. CCAs are associated with a 10–40% 5-year survival rate and show a high recurrence after surgery [3]. The purpose of this article is to summarize the current data regarding the role of endoscopic ultrasound (EUS) and EUS-tissue acquisition in the diagnostic algorithm of bile duct tumors. 

## 2. Methodology

A comprehensive search of PubMed, EMBASE, Web of Science, and the Cochrane Library databases was performed through January 2023. The following key words were applied in the search strategy: “endoscopic ultrasound” or “endosonography” and “cholangiocarcinoma” or “biliary tumor” and “staging” and “fine needle aspiration“ and “fine needle biopsy” and “contrast-enhanced endoscopic ultrasound” and “intraductal ultrasonography” and “ ERCP” and “ biliary strictures”.

## 3. Pathology

Depending on their localization, CCAs are classified into intrahepatic, perihilar, and distal [4]. Intrahepatic CCAs are located in the segmental and smaller bile ducts of the liver and account for 20% of all cholangiocarcinomas [5,6,7]. Perihilar CCAs or Klatskin tumors account for 50–60% of all cholangiocarcinomas and occur between the segmental bile ducts and at the junction between the cystic and the main hepatic duct [8,9]. Distal CCAs account for 20–30% of cases and involve the common bile duct [10].

Macroscopically, intrahepatic CCA exhibits different growth patterns; the most frequent one is mass-formation (65%), followed by periductal infiltration and intraductal growth [11,12,13]. Perihilar and distal CCAs occur most frequently as nodular plus periductal infiltrating patterns (>80%) [11,14,15]. While periductal infiltration has a longitudinally growth pattern leading to biliary strictures, intraductal tumors tend to grow towards the duct lumina [14,16].

Microscopically, the vast majority of perihilar and distal CCA are mucinous adenocarcinomas (conventional type) or papillary tumors [10,16,17], originating from the columnar mucous cholangiocytes and peribiliary glands [14,17,18,19,20,21].

Precancerous lesions include the following:

- **Biliary epithelial neoplasia** presents as flat or micropapillary lesions with dysplasia [22], sometimes associated with CCA [23]; it may occur in chronic liver disease, particularly in chronic HCV infection or alcoholic hepatitis [24]. The lesions are usually asymptomatic.
- **Intraductal papillary neoplasm (IPNBs)** can develop within intrahepatic or extrahepatic bile ducts following the classical adenoma-carcinoma sequence, have fine fibrovascular stalks, often yellow and friable, and may have a clinical and biochemical impact. In most cases, there is a high degree of dysplasia, and the epithelium from which the IPNB arises exhibits flat dysplasia [25,26]. It is possible to identify invasive CCAs in approximately half of IPNB cases, and the pancreaticobiliary subtype is more likely to be associated with invasive CCA than any other subtype [27]. In the case of IPNBs with atypia or stromal invasion, the outcomes are better compared to concurrent invasive carcinoma [28].
- **Intraductal tubulopapillary neoplasm (ITPN**) presents as a nodular mass up to 15 mm in size, with the same intraductal growth and tubular pattern as the pancreatic ITPNs [29], but with low mucin production and absent MUC5AC expression. The risk of invasive carcinoma is present in 70–80% of cases (typically tubular carcinoma [26,30]) but with a much better prognosis compared to IPNBs [26].
- **Mucinous cystic neoplasms** usually present as multilocular cysts with septation, or show a cyst-in-cyst appearance on preoperative imaging. They have a higher incidence in females and are usually diagnosed at a younger age than IPNBs, with an excellent prognosis when resected [31].

## 4. Cholangiocarcinoma Detection and Staging

EUS may be helpful in the setting of bile duct dilation if no mass is seen on CT or MRI [32], and unnecessary ERCP can be avoided in about one-third of the patients [33]. EUS evaluation of the biliary tree is performed from the level of the duodenal bulb and the distal part of the gastric antrum, for both biliary strictures and CCAs. Using EUS, they can be visualized either as a mass or as a biliary stricture.

### 4.1. Biliary Mass Detection

**Figure 1:**
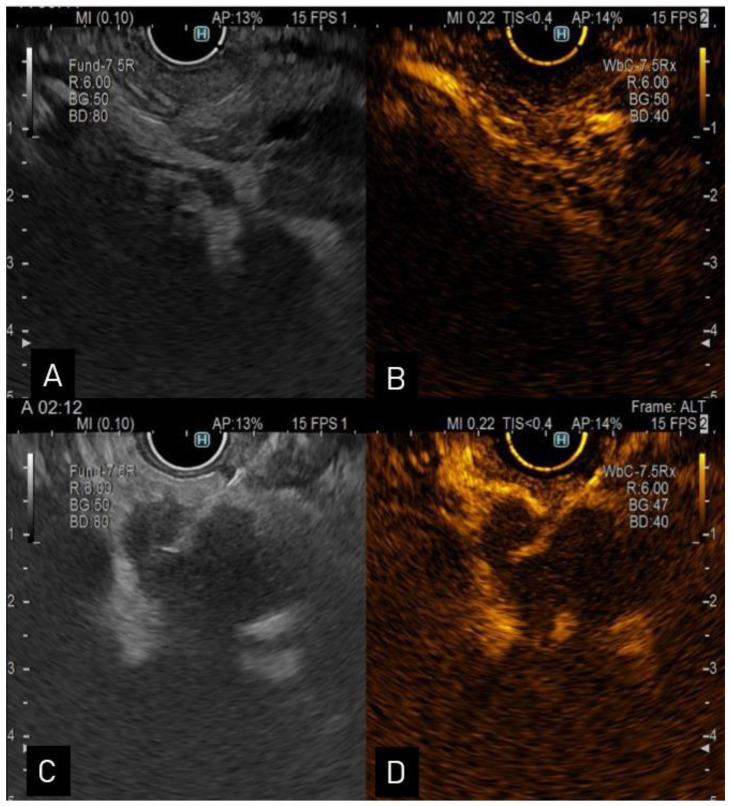
(**A**). Endoscopic ultrasound view of a distal CCA; (**B**). contrast enhanced-ultrasound showing hyperenhancement in the arterial phase; (**C**). endoscopic ultrasound view of a proximal hypoechoic bile duct lesion undergoing aspiration via a 22-G needle; (**D**). contrast enhanced-ultrasound guided aspiration.

The EUS aspect suggestive of CCA is a mass extending beyond the bile duct wall or periductal infiltration, with a wall thickness of more than 3 mm, or an intraductal mass-growing lesion [34,35] (Figure 1). In previous research, distal tumors which were closer to the EUS transducer were diagnosed in 100% of the cases, while tumors located further from the transducers were only diagnosed in 83% of the cases. Overall, EUS performed better in identifying tumors in comparison to CT or MRI (94, 30, 42%) [36]. Extrahepatic CCAs were diagnosed at an early stage when MRCP was followed by EUS (sensitivity 90% and specificity 98%) [37]. EUS is also useful when assessing common bile duct dilatation associated with normal hepatic tests and inconclusive imaging [38].

### 4.2. T Staging

EUS proved a T staging accuracy of 60–81% (Table 1), while intraductal ultrasound (IDUS) can assess the T staging with 68% accuracy [39]. No comparative data exists for T staging between proximal or distal CCAs. The growth pattern of perihilar CCAs is an axial extension along and into the bile ducts, while distal CCAs also have an axial growth pattern, extending into the pancreatic parenchyma. Invasion into the liver parenchyma is isoechoic and can easily be missed on conventional B-mode ultrasound.

For this reason, the contrast-enhanced harmonic endoscopic ultrasonography (CH-EUS) can identify the tumor as grape-like clusters that invade the biliary ducts and expand into the liver and portal vein, features useful for assessing its resectability [45]. The suggestive contrast enhanced value of CCAs is that of a hyperenhanced lesion with rapid wash-out. In a single-center retrospective study, Otsuka et al. pointed out the importance of CH-EUS for T staging in patients with perihilar CCA and distal CCA compared to surgical assessment, with a better accuracy of CH-EUS for T staging compared to conventional EUS or contrast enhanced CT (73.7% vs. 60.5 vs. 39.5%) [46]. The detection of invasion into other organs did not differ significantly between the two EUS methods (e.g., for diagnosing invasion beyond the biliary wall, the accuracy was 92.1% for CH-EUS vs. 78.9% for EUS vs. 45.9% for contrast-enhanced CT, respectively) [46]. However, CH-EUS is not recommended by the authors to be used for N or M staging, since the contrast agent is washed out rapidly [46].

In recent studies, EUS had an accuracy of 100% for identifying major vascular invasion [46], while in studies performed 10 years ago, the sensitivity for detecting unresectable tumors was only 53% [36].

No data exist about elastography during EUS assessment in extrahepatic CCAs due to the fact that these are small tumors, surrounded by many vessels, and this impedes an appropriate elastographic examination.

### 4.3. N Staging

The regional lymph nodes of the bile duct are located along the portal vein and the hepatic artery, anteriorly and posteriorly to the pancreatic head, and along the superior mesenteric artery [47]. The distant lymph nodes are located in the aortocaval, celiac, and periesophageal regions [48]. The most frequently affected lymph nodes are those in the periportal region, followed by the regional gastrohepatic sites and by the distant lymph nodes [48]. The presence of at least one malignant lymph node shortens the median survival from 1050 days to 353 [48].

The N staging accuracy varies between 66 and 81% (Table 1). It is important to keep in mind that a round, hypoechoic lesion more than 10 mm in diameter is not specific for malignancy [40]. EUS can identify nodal involvement with a higher accuracy than cross-sectional imaging (86 vs. 47%) [48]. The presence of malignant regional lymph nodes precludes curative oncological resection or liver transplant for CCA [49], and is associated with a four-fold higher risk of death [48].

## 5. Cholangiocarcinoma Presents as Strictures

Extrahepatic CCA usually presents as biliary strictures. Up to 24% of proximal biliary strictures are benign (IgG4 cholangiopathy, primary sclerosing cholangitis, eosinophilic cholangitis, biliary papillomatosis, response to infection, trauma, ischaemia) [50], while 70–80% can be caused by malignancies (cholangiocarcinoma, bile-duct lymphoma, gallbladder carcinoma or metastatic disease) [34,51,52]. In the case of a distal stricture, the differential diagnosis should be made from pancreatic cancer, invasive IPMN, chronic pancreatitis, or ampullary lesions. It is crucial to differentiate histologically between the benign and malignant strictures in order to avoid unnecessary surgery. In previous studies, EUS has a sensitivity of over 86% for assessing bile duct stenoses, with increased accuracy for lesions in the distal part of the common bile duct (CBD) [50].

EUS can identify and provide additional information about biliary strictures, without etiology confirmed by other imaging modalities [50]. EUS can specify their location (distal/medium/proximal common bile duct) and their malignant characteristics, and may detect and stage tumors [53]. Biliary stents should be retrieved before performing EUS in order to obtain an appropriate CBD assessment.

There are only 13 articles assessing CCAs presented as strictures (Table 2). EUS criteria for a malignant stricture are: the disruption of the trilaminar bile duct wall, a hypoechoic mass of more than 5 mm, or a wall thickness of more than 3 mm with an irregular outer edge of the bile duct [54]. EUS has a sensitivity of 79–93% and a specificity of 94–97% in diagnosing malignant biliary strictures; after excluding a possible extrinsic compression, the diagnostic yield reached 79% to 99%, offering a suitable examination of the extrahepatic biliary tree [55]. In previous research, EUS showed a sensitivity of 79–89% for distal CCAs and 57–68% for perihilar CCAs presenting as strictures [56,57]. In a retrospective study, the accuracy of detecting malignant strictures by EUS appeared to be similar to CT (70 vs. 79%) [57].

## 6. EUS Tissue Acquisition of Mass-Forming Cholangiocarcinoma

Current guidelines recommend core needle biopsy for unresectable tumors or metastatic CCAs [32]. Criteria for defining an unresectable distal CCA include major vessel involvement (superior mesenteric artery) and distant metastases and lymph node metastases beyond the portal vein, hepatic artery, peripancreatic, or celiac trunk. Unresectable perihilar CCA criteria are: distant metastases and lymph node involvement (similar to distal CCA), contralateral or bilateral major vessel involvement (similar to distal CCA), and inadequate future liver remnant volume [69].

Theoretically, there is a risk of tumor peritoneal seeding when performing EUS fine-needle aspiration (EUS-FNA) in a transperitoneal manner, as described in a study with 13 percutaneous FNA and 3 EUS-FNA [70]. In this study peritoneal spreading occurred in 5 of 6 patients with positive FNA for malignancy, limiting the possibility of the patient benefiting from a curative surgery [70,71]. However, EUS-FNA is usually performed from the duodenal bulb in the retroperitoneum, and the potential spreading foci would be removed during surgery. EUS-FNA is not considered an absolute contraindication for curative surgery, especially in distal CCA. Moreover, a retrospective study showed that preoperative EUS-FNA does not affect survival or progression-free survival of patients with CCA [72], but no prospective study exists regarding this issue.

Technically, EUS-FNA is performed in the long position of the endoscope, usually from the duodenal bulb. Detecting and sampling hilar tumors (44–83% sensitivity) is more difficult than for identifying tumors, as they are farther from the transducer, smaller, and more often infiltrative [73] (Table 3). Mass-forming CCAs are easier to sample through EUS than stricture-forming CCAs, and the main reason for a false negative result of EUS-FNA may be related to the intraepithelial superficial spread; in such a situation ERCP sampling could be helpful, preferably with cholangioscopy [74].

The architecture of the tissue is not preserved with EUS-FNA needles, and for this reason, EUS-FNB needles are preferred. They are more extensively used in pancreatic pathology than in biliary tumors. Currently, there are no conclusive data regarding the use of EUS-FNB in CCA. A study by Troncone et al. pointed out that EUS-FNA/FNB has a sensitivity, specificity, and diagnostic accuracy of 73.9, 100, and 80%, respectively [75]. For distal biliary stenosis, the sensitivity and accuracy of EUS-FNA/B were 44.4 and 64.3% respectively, increasing to 90.1 and 91.7% in the case of proximal biliary stenosis [75]. As found in a retrospective study, the size of needles (22 vs. 25G vs. 19G) did not influence the diagnostic rate (70.9 vs. 75.3 vs. 66.7%), and complications such as bleeding have been rarely reported [36,78]. Only one study used rapid on-site evaluation during EUS-FNA, and the results were similar to those of other studies [81].

Biliary stents can lower the diagnostic accuracy of EUS-FNA from 95 to 65% in distal lesions, and from 86 to 56% in perihilar lesions because the access of the needle beyond the stent is restricted [76]. For this reason, it is recommended to remove the stent prior to EUS tissue acquisition, similar to pancreatic pathology, in which the diagnostic value of EUS-FNB is diminished by the presence of a biliary stent, regardless of its type [82]. Moreover, in the case of primary sclerosing cholangitis, the presence of multiple biliary stenosis and benign lymphadenopathy may make EUS-FNA more difficult [56,76].

## 7. EUS Tissue Acquisition from Lymph Nodes

In a retrospective series of 157 CCAs staged with cross-sectional imaging, EUS, and/or laparotomy, there were 31 malignant lymph nodes, proved by EUS-FNA in 87% and overlooked by cross-sectional imaging in 39% of them [48]. However, there is a lack of evidence related to the risk of malignant seeding during EUS-FNA, as shown previously. In cases of potential resectable CCAs, lymph node sampling should be performed only if it changes the surgical decision [83].

## 8. EUS Tissue Acquisition vs. ERCP Sampling

ERCP sampling for mass-forming CCAs required three biopsies, and multiple biopsies in case of stricture form CCAs [74].

Given that ERCP has been shown to have limited diagnostic power in detecting malignancy, either by brushing, biopsy, or cholangioscopy alone [84], or by combining brushing and biopsy [85], a comparison between methods was considered necessary. A meta-analysis of data from 422 patients in 17 studies showed that EUS-FNA had a higher sensitivity (73.6%, 95% CI 64.7–81.5%, I^2^ = 74.7%) than biopsy alone (67%), brushing alone (56%), or brushing plus biopsy (70%) [86].

While Onoyama et al. did not observe any significant differences in the diagnostic value of ERCP vs. EUS-FNA for patients with distal biliary lesions, with a better outcome only when comparing the side events (25.9 vs. 0%) [77], other studies pointed out the superiority of EUS-FNA to ERCP, reporting a 59% sensitivity in negative brush cytology patients [85] (Table 4).

## 9. EUS Tissue Acquisition of Biliary Strictures

In order to obtain a definitive diagnosis, tissue acquisition is important, and this could be very difficult due to mucosal superficial spread, especially in proximal tumors, a situation in which EUS-FNA has limited value (25–89% for proximal strictures and 43–100% for all strictures) (Table 2). For this reason, according to the ESGE guidelines, EUS sampling is preferred for distal and extraductal lesions [91]. In a relatively recent meta-analysis, the diagnostic sensitivity of biliary strictures of EUS-FNA was superior to ERCP sampling (75 vs. 49%), especially in cases of extraductal lesions (100 vs. 54.8%) [92]. According to the ESGE guideline, ERCP sampling (intraductal biliary brushing and/or forceps biopsy) represents the first choice for proximal and wall thickening CCAs [91]. After prior ERCP sampling, tissue can be obtained by EUS-FNA [51]. Since EUS-FNA exhibits low negative predictive values (34–47%), a negative result cannot exclude a tumoral etiology of the stricture [92,93], and repeated EUS-FNA is indicated, although the additional sensitivity of the second EUS-FNA is low (only 17%) [76]. The presence of a mass on EUS increases the sensitivity of tissue acquisition compared to that of wall thickening tumors [35].

In a meta-analysis including 497 patients with biliary strictures who underwent EUS and ERCP in the same session, EUS-guided tissue sampling showed better diagnostic accuracy in comparison with ERCP (94.5 vs. 78.1%). When both were used, the sensitivity, specificity, positive likelihood ratio, negative likelihood ratio, and accuracy were 86, 98, 12.50, 0.17, and 96.5%, respectively [90].

When possible, both EUS and cholangioscopy should be used [94,95]. The use of cholangioscopy in biliary strictures demonstrated a 60% (38–88%) sensitivity compared to the EUS-FNA of 80% (46–100%) [51], but the use of cholangioscopy after EUS-FNA may obtain the correct clinical diagnostic in 94% of the cases, resulting in cost savings [41].

The fluorescence in situ hibridization (FISH) technique may increase the sensitivity of cytology in distal and proximal strictures from 33 to 93% (*p* < 0.001) and from 48 to 76% (*p* = 0.05), respectively [42]. Genetic alterations can be identified in EUS-FNA samples from the biliary tract, and perhaps this technique will continue to be developed in the future [43]. Moreover, portal vein sampling for identifying tumor cells or circulating tumor DNA, RNA, exosomes, cytokines, and proteins could provide supplementary information in these patients [44]. However, this technique is still experimental, and further studies are required for establishing its potential role [96].

## 10. Intraductal Ultrasonography for Biliary Strictures

Intraductal ultrasonography (IDUS) can evaluate the biliary tract and its normal trilaminar structure with a proximal hyperechoic mucosal layer, followed by a middle hypoechoic fibromuscular layer and a distal hyperechoic subserosa layer. Malignant features on IDUS include disruption in the bile duct layering by a hypoechoic sessile mass with irregular borders and the presence of lymphadenopathy [97]. Malignant strictures can present as a nodule of more than 8 mm in size (PPV of 100%), even if the wall ultrasound architecture is intact. While EUS is more accurate in detecting and diagnosing distal bile duct tumors [98], IDUS has higher accuracy for proximal lesions [99]. IDUS has higher sensitivity, specificity, and accuracy when compared to ERCP (87.5, 90.6, and 90%, respectively vs. 62.5, 53.1, and 55%, respectively) [100]. When the two methods are combined, the diagnostic power is over 92–93% [57]. IDUS seems to be better than EUS in both T staging and predicting tumor resectability (sensitivity 89.1 and 81.8 vs. 75.6% and 75.6%, respectively; *p* < 0.002), and similar for N staging (accuracy 60 vs. 62.5%) [73]. As IDUS alone cannot be used to obtain tissue samples, IDUS-guided transpapillary forceps biopsy has a higher malignancy detection rate than biopsy under ERCP for infiltrating CCAs (90.8 vs. 76.9%) [73,101].

## 11. Diagnostic Algorithm

In cases of distal strictures or masses identified by cross sectional imaging, EUS and EUS tissue acquisition should be performed before ERCP. In the case of a proximal strictures or mass identified by cross sectional imaging, EUS could be performed as an adjunctive to ERCP when this is inconclusive. However, in the case of resectable tumors, only EUS staging should be performed. By contrast, core-needle biopsy tissue acquisition should be performed only in unresectable and metastatic CCAs [49,102].

## 12. Conclusions

This study summarizes the available evidence regarding the role of EUS in diagnosing and staging extrahepatic CCAs. With superior diagnostic value compared to brushing and biopsy during ERCP, EUS tissue acquisition is better when assessing malignant biliary lesions, either unresectable proximal biliary lesions or distal resectable and unresectable lesions. Despite this, a negative result does not exclude malignancy, as both methods show a low negative predictive value, and the EUS tissue acquisition should be repeated. Perhaps the development of an EUS-fine needle biopsy would increase the diagnostic yield in such lesions.

## Figures and Tables

**Table 1:** Accuracy of endoscopic ultrasound staging in extrahepatic cholangiocarcinoma.

Author, Year	No. of Patients (*n*)	Type of Study	Accuracy of T Staging (%)	Accuracy of N Staging (%)	Accuracy of Portal Vein Invasion (%)	Accuracy of Hepatic Artery Invasion (%)
Otsuka 2022, [40]	38 (22 were T1 and T2 cases)	R ^1^	CH-EUS ^3^ 73.7 EUS ^4^ 60.5	-	CH-EUS 100 EUS 100	CH-EUS 100 EUS 100
Malikowski 2020, [41]	133	R	-	86	-	-
Sugiyama 1999, [42]	19	P ^2^	-	-	100	-
Tio 1993, [43]	46	R	66	64	-	-
Mukai 1992, [44]	16	R	81	81	88	-

^1^ R—retrospective; ^2^ P—prospective; ^3^ CH-EUS—contrast-enhanced harmonic endoscopic ultrasonography; ^4^ EUS—endoscopic ultrasound.

**Table 2:** Diagnostic value of endoscopic ultrasound fine needle aspiration of biliary strictures.

Author, Year	No of Pts/No of Proximal Strictures, *n*/*n*	Type of Study	Type of FNA Needle	Diagnostic Value of EUS-FNA %	Diagnostic Value of EUS-FNA for Proximal Strictures %
Lee 2019, [58]	27/0	P ^1^	FNA ^3^ 22,25G	96.3	-
Yeo 2019, [59]	93/0	R ^2^	FNA 19,20,22,25G	86.8	-
Weilert 2014, [60]	15/8	P	FNA 22,25G	80	-
Nayar 2011, [61]	32/32	R	FNA *	52	52
Ohshima 2011, [62]	22/9	R	FNA 19,22,25G	100	-
Novis 2010, [63]	11/3	P	FNA 22G	69.4	-
DeWitt 2006, [50]	24/24	P	FNA 22G	-	Sn ^4^ = 77, Sp ^5^ = 100, PPV ^6^ = 100, NPV ^7^ = 29
Byrne 2004, [64]	35/3	R	FNA 22G	45–100	-
Eloubeidi 2004, [65]	28/15	P	FNA 22G	86	-
Fritscher Ravens 2004, [66]	44/44	P	FNA 22G	-	89
Lee 2004, [35]	42/1	R	FNA *	47	-
Rosch 2004, [67]	28/11	P	FNA 22G	43	25
Fritscher Ravens 2000, [68]	10/10	P	FNA 22G	-	89

^1^ P—prospective; ^2^ R—retrospective; ^3^ FNA—fine needle aspiration; ^4^ Sn—sensitivity; ^5^ Sp—specificity; ^6^ PPV—positive predictive value; ^7^ NPV—negative predictive value; * needle size unreported.

**Table 3:** The diagnostic value of endoscopic ultrasound tissue acquisition in cholangiocarcinoma.

Author, Year	No. of Pts	Type of Study	Type of Needle	Final Diagnosis	Sn ^7^ %	Sp ^8^ %	PPV ^9^ %	NPV ^10^ %	Acc ^11^ %
Troncone 2022, [75]	29	R ^1^	FNA ^3^/ FNB ^4^ 22G	ERCP ^6^ or EUS or surgery	Distal 44.4 Proximal 90.1	Distal 100 Proximal 100	-	-	Distal 64.3 Proximal 91.7
Raine 2020, [76]	80	R	FNA 22G	surgery	77	100	100	60	-
Onoyama 2019, [77]	37	R	FNA 22G	EUS-FNA or ERCP	81.8	87.5	90	77.8	84.2
Jo 2018, [78]	53	R	FNA 22, 25, 19G	surgery or EUS-FNA and/or ERCP or follow-up	75	-	-	18.1	76.3
Onda 2016, [56]	37	P ^2^	FNA 22G	37 EUS-FNA	84	100	100	63	87
Weilert 2014, [60]	13	P	FNA 22, 25G	surgery or EUS-FNA and/or ERCP or follow-up	79	-	-	-	80
Tummala 2013, [79]	28	R	FNA *	EUS	91.5	94.6	97.8	80.9	92.4
Krishna 2012, [80]	18	P	FNA 22, 25G	Surgery or EUS or follow-up	66.6	100	100	62.5	78.6
Mohamadnejad 2011, [36]	74	P	FNA 22G	surgery	Distal 81 Proximal 59	-	-	-	-
Meara 2006, [81]	44	P	FNA 22G + ROSE ^5^	EUS-FNA+ follow-up	87	100	-	-	-

^1^ R—retrospective; ^2^ P—prospective; ^3^ FNA—fine-needle aspiration; ^4^ FNB—fine-needle biopsy; ^5^ ROSE—rapid on-site evaluation; ^6^ ERCP—endoscopic retrograde cholangiopancreatography; ^7^ Sn—sensitivity; ^8^ Sp—specificity; ^9^ PPV—positive predictive value; ^10^ NPV—negative predictive value; ^11^ Acc—accuracy. * Needle size unreported.

**Table 4:** Comparative diagnostic value of endoscopic ultrasound and ERCP tissue acquisition for biliary tumors.

Author, Year	Type of Lesions	Type of Study	Diagnostic Value of EUS-FNA ^4^	Diagnostic Value of ERCP ^10^ Biopsy
Mattheu 2022, [87]	77 biliary obstruction	R ^2^	Se ^5^ 90.6%, Acc ^6^ 92.6%	Se 65.6%, Acc 71.4%
Trocone 2022, [75]	29 CCA ^1^ 18 benign	R	Se 73.9%, Sp ^7^ 100%, Acc 80%	Se 66.7%, Sp 100%, Acc 80%
Chung 2021, [88]	70 CCA 1 metastasis 14 benign	R	Se 80.3%, Acc 83.5%	Se 67.6%, Acc 72.9%
Yang 2021, [89]	307 malignant (136 CCA) 169 benign	R	Se 44.4%, Sp 100%, PPV ^8^ 100%, NPV ^9^ 28.6%, Acc 54.6%	Se 61.1%, Sp 100%, PPV 100%, NPV 56.3%, Acc 74.1%
Jo 2019, [78]	53 CCA	R	Se 75%, Acc 76.3%	Se 73.6%, Acc 75%
Onoyama 2019, [77]	37 CCA 36 benign	R	Se 81.8%, Sp 87.5%, PPV 90%, NPV 77.8%, Acc 84.2%	Se 76.0%, Sp 100%, PPV 100%, NPV 82%, Acc 88%
Yeo 2019, [59]	86 malignant (39 CCA) 7 benign	R	Se 86.8%, Sp 100%, Acc 87.8%, PPV 100%, NPV 37.5%	Se 78.9%, Sp 100%, Acc 80.5%, PPV 100%, NPV 27.3%
Moura 2018, [90]	47 malignant 1 suspicious 2 benign	P ^3^	Se 93.8%, Ac 94%	Se 60.4%, Ac 62%
Heinzow 2014, [57]	56 CCA	R	Se 57%, Sp 78%, Acc 70%	Se 96%, Sp 89%, Acc 92%
Weilert 2014, [60]	48 malignant (14 CCA) 3 benign	P	Se 79%, Ac 94%	Se 79%, Ac 53%

^1^ CCA—cholangiocarcinoma; ^2^ R—retrospective; ^3^ P—prospective; ^4^ EUS-FNA—endoscopic ultrasound fine needle aspiration; ^5^ Se—sensitivity; ^6^ Acc—accuracy; ^7^ Sp—specificity; ^8^ PPV—positive predictive value; ^9^ NPV—negative predictive value; ^10^ ERCP—endoscopic retrograde cholangio-pancreatography.

## Data Availability

The study did not report on any new data.
